# ﻿*Rhizophorastylosa* (Rhizophoraceae) newly recorded from Thailand: lectotypification, leaf anatomy, and pollen morphology

**DOI:** 10.3897/phytokeys.259.157847

**Published:** 2025-06-17

**Authors:** Chatchai Ngernsaengsaruay, Nittaya Mianmit, Supol Kamsanor, Rachanee Pothitan, Vipak Jintana, Nirat Jintana, Pichet Chanton, Apinya Sukantatul, Jutiporn Khade

**Affiliations:** 1 Department of Botany, Faculty of Science, Kasetsart University, Chatuchak, Bangkok 10900, Thailand; 2 Biodiversity Center, Kasetsart University (BDCKU), Chatuchak, Bangkok 10900, Thailand; 3 Department of Forest Management, Faculty of Forestry, Kasetsart University, Chatuchak, Bangkok, 10900, Thailand; 4 Mu Ko Phetra National Park, Department of National Parks, Wildlife and Plant Conservation, Pak Nam Subdistrict, La-Ngu District, Satun Province, 91110, Thailand; 5 Department of National Parks, Wildlife and Plant Conservation, Chatuchak, Bangkok, 10900, Thailand; 6 Suan Luang Rama IX Foundation, Nong Bon Subdistrict, Prawet District, Bangkok, 10250, Thailand

**Keywords:** Lectotypification, Malpighiales, morphology, palynology, plant anatomy, red mangrove, Rhizophoreae, taxonomy, true mangrove tree, vivipary

## Abstract

*Rhizophorastylosa* (Rhizophoraceae), previously known from India (Andaman and Nicobar Islands), China to Indo-China, and the Pacific Island, is reported here as a new record from coastal areas with muddy-sandy, sandy or sandy-rocky substrates at Ko Lidi and Ko Bulon Le, within Mu Ko Phetra National Park, La-ngu District, and at Ao Talo Wao, Ko Tarutao, Tarutao National Park, Mueang Satun District, Satun Province, Peninsular Thailand. A detailed morphological description, illustrations and a distribution map of the species are provided, along with notes on distribution, habitat and ecology, phenology, a preliminary conservation assessment, etymology and specimens examined. In addition, *Rhizophorastylosa* is lectotypified, the Thai vernacular name “Kongkang phetra” is herein proposed for this species, and an updated identification key to the species of *Rhizophora* in Thailand is presented. The species is currently assessed as Least Concern [LC] under the IUCN conservation status. Furthermore, detailed descriptions of leaf anatomical features and pollen morphology are provided. The thick cuticles, sunken stomata, large hypodermal cells, and cork warts are adaptive anatomical features of leaves in *Rhizophora* that live in the mangrove environments. The pollen grains of *Rhizophorastylosa* are tricolporate, prolate spheroidal or oblate spheroidal shapes, small-sized, and reticulate exine sculpturing.

## ﻿Introduction

Rhizophoraceae Pers. is well known as the most species-rich mangrove family, comprising four exclusively mangrove genera and 19 species: *Bruguiera* Lam. ex Savigny (six species and two natural hybrids), *Ceriops* Arn. (five species), *Kandelia* (DC.) Wight & Arn. (two species), and *Rhizophora* L. (six species and five natural hybrids) (Tomlinson 1986, 2016; Sheue et al. 2012; POWO 2025; WFO 2025). All members of the Rhizophoraceae have large, conspicuous, glabrous and caducous interpetiolar stipules on both vegetative and reproductive shoots, which strongly ensheath the developing leaves and inflorescences (Hou 1958). In species of mangrove Rhizophoraceae, several to hundreds of aggregated finger-like colleters mixed with milky mucilage can be observed with the naked eye at the adaxial base of the stipules (Tomlinson 1986, 2016; Sheue 2003). The structural and mechanical protection provided by stipules and colleter exudates may help shield the young shoots of these mangrove plants from their harsh environmental conditions (Sheue et al. 2012).

The tribe Rhizophoreae Bartl. is characterized by the following features: true mangrove shrubs or trees, with aerial roots; glabrous and caducous interpetiolar stipules that sheath the terminal bud and young leaves; decussate, entire leaves; cymose inflorescences; 4–16-merous flowers; mostly diplostemonous androecia (with twice as many stamens as petals); half-inferior to fully inferior ovaries, 2–3-carpellate with two ovules per carpel; baccate, fibrous fruits usually containing a single seed; and viviparous germination. This pantropical tribe comprises four genera: *Bruguiera*, *Ceriops*, *Kandelia*, and *Rhizophora* (Henslow 1878; King 1897; Hou 1958; Schwarzbach 2014).

*Rhizophora* is a group of tropical and subtropical mangrove trees, commonly referred to as true mangroves. It is a small genus belonging to the order Malpighiales Juss. ex Bercht. & J. Presl, the family Rhizophoraceae (The Angiosperm Phylogeny Group 2016), and the tribe Rhizophoreae (Schwarzbach 2014). Eleven taxa have been recognized within the genus, comprising six accepted species and five natural hybrids. Among these, two species and one hybrid occur in the Americas; three species and one hybrid in Africa; three species and two hybrids in the Indian subcontinent, China, and Southeast Asia (POWO 2025); three species and one hybrid in Australia (Duke and Bunt 1979; McCusker 1984; POWO 2025); and four species and three hybrids in the Pacific region (Melanesia, Micronesia, and Polynesia) (Tomlinson 1978; POWO 2025). These three species of *Rhizophora* found in the Indian subcontinent, China, and Southeast Asian species are *R.apiculata* Blume, *R.mucronata* Poir., and *R.stylosa* Griff. (Hou 1958, 1960; Qin and Boufford 2007; Marchand 2008; POWO 2025). In addition, two natural hybrids occur in the region: R.×annamalayana Kathiresan (*R.apiculata* × *R.mucronata*) and R.×lamarckii Montrouz. (*R.apiculata* × *R.stylosa*) (Parani et al. 1997; Ng and Chan 2012; Ng et al. 2013; Setyawan et al. 2014; Ragavan et al. 2015; POWO 2025).

Previously, Hou (1970) treated the genus *Rhizophora* in the “Flora of Thailand”, recognizing two species: *R.apiculata* and *R.mucronata*. However, this account was brief, providing limited morphological descriptions and lacking other supporting information. Recently, Ngernsaengsaruay et al. (2024) revised the genus *Rhizophora* for Thailand, offering updated and detailed morphological descriptions, an identification key, and comprehensive notes on distribution, habitat and ecology, phenology, conservation assessments, and lectotypifications. In addition, leaf anatomical characters and pollen morphology were examined to provide a complete overview of *Rhizophora* in Thailand.

In 2007, Mr.Nirat Jintana and Associate Professor Dr.Vipak Jintana discovered a plant belonging to the genus *Rhizophora* on Ko Lidi and Ko Bulon Le, within Mu Ko Phetra National Park, La-ngu District, Satun Province. However, no specimens were collected at the time, and the species remain unidentified. During fieldwork conducted at the same locations in April 2025, and at Ao Talo Wao, Ko Tarutao, Tarutao National Park, Mueang Satun District, Satun Province in May 2025, we collected specimens bearing both vegetative and reproductive parts. These specimens match the type and descriptions found in various taxonomic references of *Rhizophorastylosa* Griff. (e.g., Griffith 1854; Hou 1958; Backer and Bakhuizen van den Brink 1963; Duke and Bunt 1979), a species previously recorded from India, China to Indo-China, and the Pacific, but not Thailand. Consequently, this represents a new record for Thailand. A detailed morphological description, illustrations, lectotypification, and an updated identification key to the species of *Rhizophora* in Thailand are provided, along with notes on distribution, habitat and ecology, phenology, preliminary conservation assessment, etymology, vernacular name and specimens examined. Additionally, leaf anatomical characteristics and pollen morphology are presented.

## ﻿Materials and methods

Plant specimens of *Rhizophorastylosa* were observed and collected in the peninsular region of Thailand. The collected specimens were identified by consulting taxonomic literature (e.g., Griffith 1854; Henslow 1878; Ridley 1922; Hou 1958, 1960; Backer and Bakhuizen van den Brink 1963; Tomlinson 1978; Duke and Bunt 1979; McCusker 1984; Qin and Boufford 2007; Duke 2010; Ng and Chan 2012; Setyawan et al. 2014; Ragavan 2015; Ragavan et al. 2015, 2021; Kannan et al. 2021) and by comparison with herbarium specimens housed in the following herbaria: BK, BKF, as well as those available through the virtual herbarium databases of A, BR, CAL, E, K, L, P, SING, US, and GBIF (https://www.gbif.org/). All herbarium codes follow Thiers (2025, continuously updated). The taxonomic history of the species was compiled from both published literature and online databases (IPNI 2025; POWO 2025; WFO 2025). Morphological characters, distribution, habitat and ecology, phenology, and uses were described based on newly collected herbarium specimens and the author’s field observations. The distribution map was generated using the R program, based on data from the specimen examined and credible literature sources (e.g., Ragavan 2015; Kannan et al. 2021; Ragavan et al. 2021). Conservation status was assessed according to the IUCN Red List Categories and Criteria (IUCN Standards and Petitions Committee 2024), supplemented with GeoCAT analysis (Bachman et al. 2011) and field data.

The leaf anatomical features of *Rhizophorastylosa* were investigated through transverse sectioning using a sliding microtome at thickness of 15–20 µm. To study the epidermal cells, the leaves were peeled and mounted. Permanent slides were prepared according to the standard methods described by Johansen (1940) and Kermanee (2008). Anatomical characteristics were observed and recorded photographically using an Olympus BX53 microscope equipped with an Olympus DP74 digital camera at the Department of Botany, Faculty of Science, Kasetsart University (KU). Anatomical terminology follows the conventions outlined by Metcalfe and Chalk (1957).

Samples of pollen grains were examined and recorded photographically using an Olympus BX53 microscope equipped with an Olympus DP74 digital camera. For scanning electron microscopy (SEM), materials were prepared at the Scientific Equipment Centre, Faculty of Science, Kasetsart University, by mounting the pollen grains on stubs with double-sided sellotape, sputter-coating them with gold, and examining them using an FEI Quanta 450 SEM (Hillsboro, OR, USA) at 15.00 kV. The characteristics of the pollen grains were examined and measured following the methods of Erdtman (1945, 1952) and Simpson (2010). Pollen morphology terminology follows that of Punt et al. (2007).

## ﻿Results and discussion

### ﻿Taxonomic treatment

#### ﻿ Rhizophora stylosa

Taxon classificationPlantaeMalpighialesRhizophoraceae

Griff., Not. Pl. Asiat. 4: 665. 1854; G. Hensl. in Hook. f., Fl. Brit. India 2: 436. 1878; Ridl., Fl. Malay Penins. 1: 693. 1922; Ding Hou, Fl. Males., Ser. 1, Spermat. 5(4): 456, figs. 5, 8a–f, 13. 1958 et Blumea 10(2): 629. 1960; Backer & Bakh. f., Fl. Java 1: 380. 1963; Toml., J. Arnold Arbor. 59: 163, figs. 3, 4D–F. 1978; N. C. Duke & J. S. Bunt, Aust. J. Bot. 27: 672. fig. 6a–b. 1979; A. McCusker in A. S. George et al., Fl. Australia 22: 2. 1984; W. Giesen et al., Mangrove Guideb. S.E. Asia: 239, fig. 239. 2007; H. Qin & Boufford, Fl. China 13: 296, fig. 316(1–9). 2007; N. C. Duke, Blumea 55: 179, fig. 8. 2010; A. D. Setyawan et al., Nusantara Bioscience 6(1): 77, figs. 6A.b–G.b, 8. 2014; Ragavan, Taxon. Mangroves Andaman and Nicobar Isl. PhD Thesis: 115, fig. 43. 2015; Ragavan et al., Indian Mangroves: 122–123. 2021; S. Kannan et al., Bot. Mar. 64(3): 7–8, figs. 3D, 5B, D, F, G, J, L, M (right), O, 2021.

BF9B24D7-9F6E-58BC-B854-545544BDE3D6

**Figure 1.**
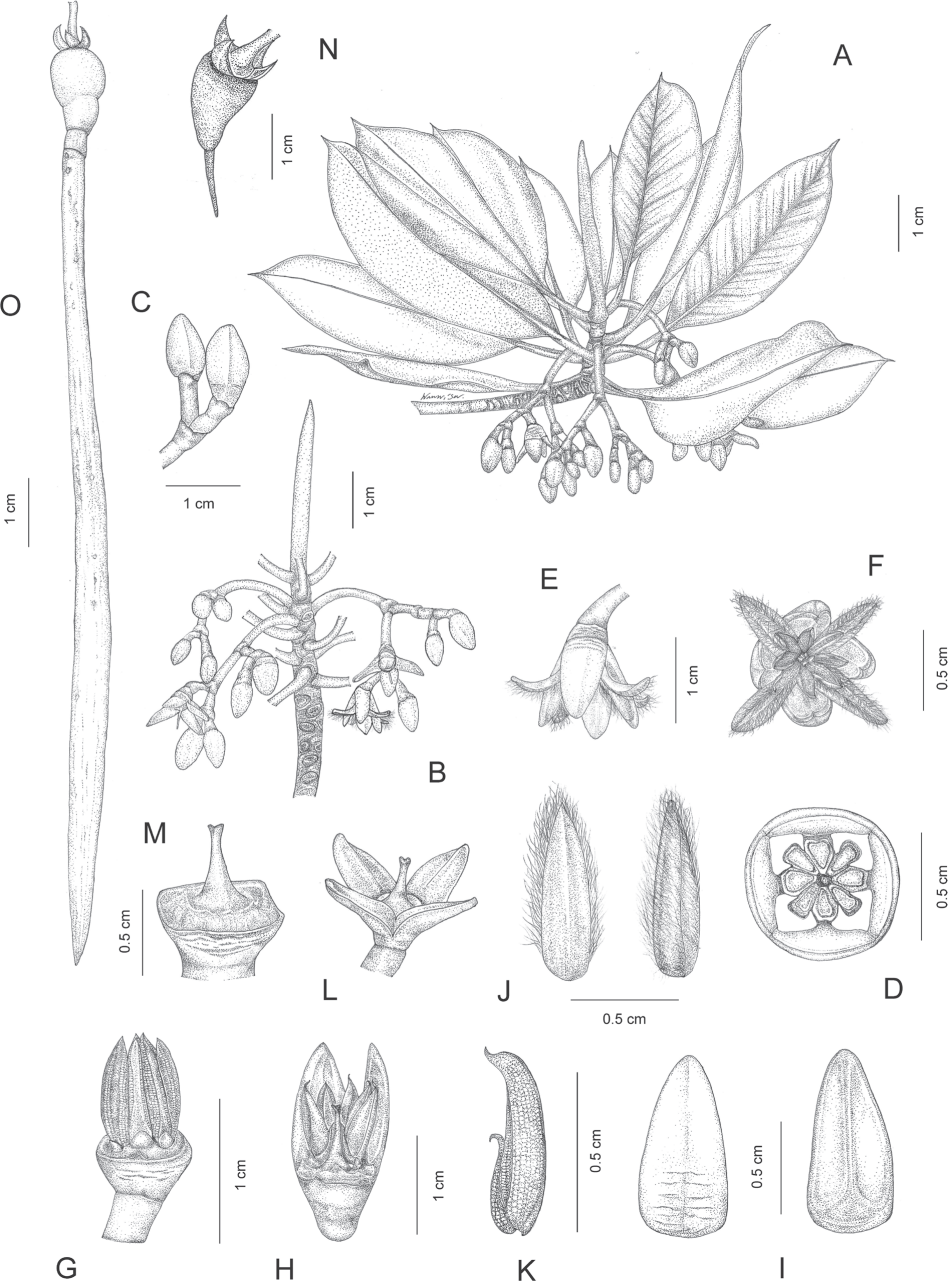
*Rhizophorastylosa*. **A, B.** Flowering branchlets showing a terminal interpetiolar stipule, leaves, and inflorescences with flower buds and fully open flowers; **C.** Flower buds; **D.** Flower bud in transverse section; **E.** Fully open flower in lateral view; **F.** Fully open flower in top view; **G.** Flower bud showing stamens with sepals and petals removed; **H.** Flower bud in longitudinal section; **I.** Sepals; **J.** Petals with densely long-villous along margins; **K.** Stamen; **L–M.** Pistils showing a long style; **N–O.** Fruits showing successive stages in development of hypocotyl. Drawn by Wanwisa Bhuchaisri.

**Figure 2.**
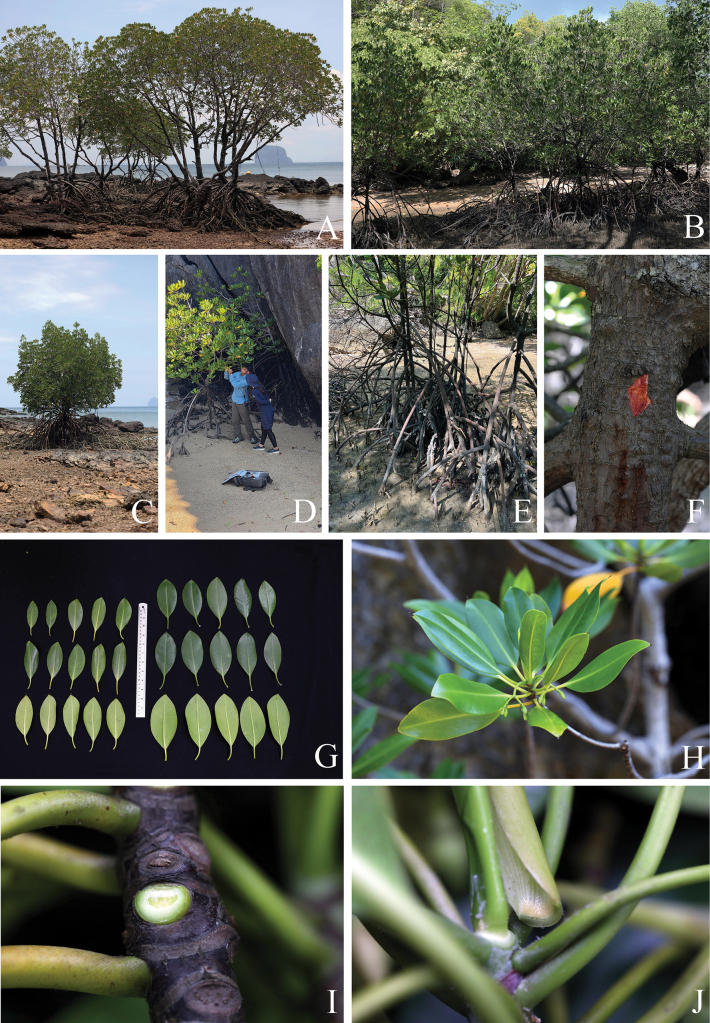
*Rhizophorastylosa*. **A–D.** Habitats and habit; **E.** Stems supported by numerous branched stilt roots; **F.** Stem, outer bark and inner bark; **G.** Leaves showing adaxial and abaxial surfaces: Population A (left) and Population B (right); **H.** Branchlets, leaves and young inflorescences; **I.** Leaf scar with many tiny vascular bundle strands, arranged in a crescent moon; **J.** Stipule with dense colleters aggregated in a basal band, producing a sticky white exudate. Photos: Chatchai Ngernsaengsaruay (**A–C, E–J**) and Rachanee Pothitan (**D**).

**Figure 3.**
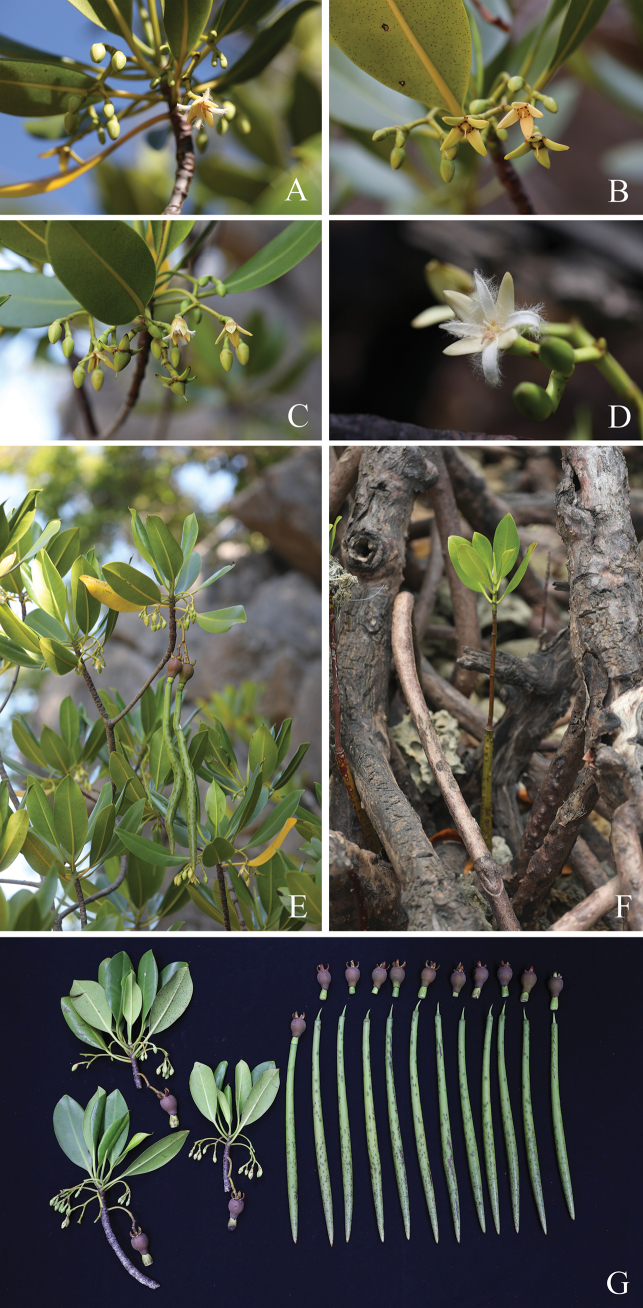
*Rhizophorastylosa*. **A, B.** Flowering branchlets showing inflorescences with flower buds and fully open flowers; **C.** Flowering branchlets showing flower buds and fully open flowers and fruiting branchlet showing young fruits; **D.** Inflorescence with flower buds and fully open flower; **E, G.** Flowering and fruiting branchlets showing leaves, inflorescences with flower buds and fully open flowers, and hypocotyls. **F.** Seedling. Photos: Chatchai Ngernsaengsaruay.

**Figure 4.**
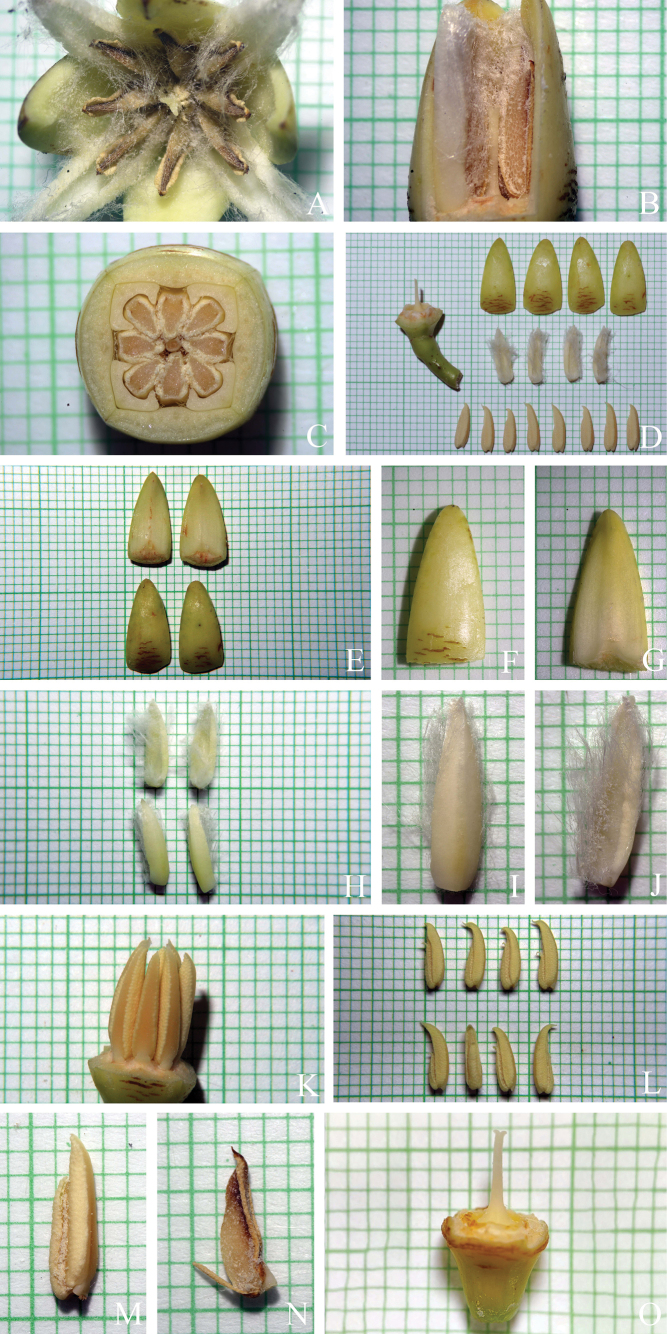
*Rhizophorastylosa*. **A.** Fully open flower; **B.** Flower bud in longitudinal section; **C.** Flower bud in transverse section; **D.** Dissected flower showing sepals, petals, stamens, and pistil; **E–G.** Sepals; **H–J.** Petals with densely long-villous along margins; **K–N.** Stamens; **O.** Pistil showing a long style. Photos: Pichet Chanton (**A–N**), Apinya Sukantatul (**O**).

**Figure 5.**
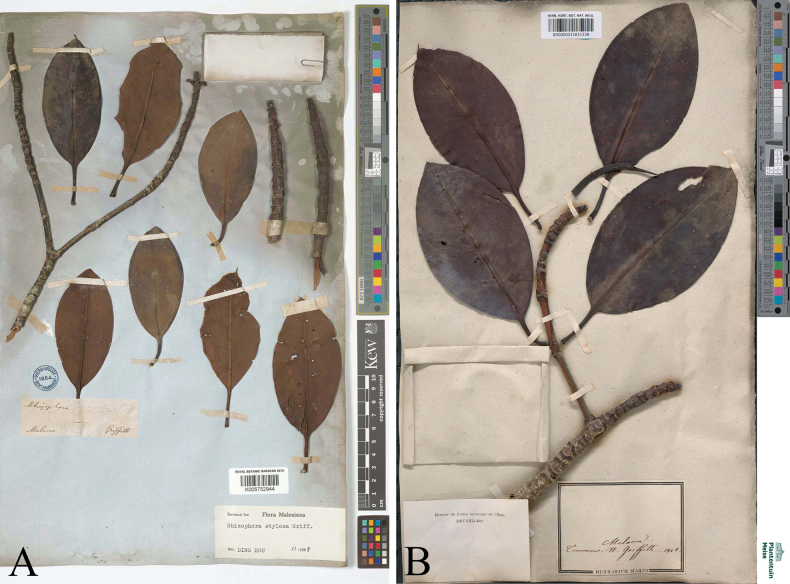
Lectotype and isolectotype of *Rhizophorastylosa* from Malacca, Peninsular Malaysia. **A.** Lectotype, *W. Griffth s.n.* (K [K005752944]); **B.** Isolectotype, *W. Griffth s.n.* (BR [BR0000031831338]). Photos: © The Board of Trustees of the RBG, Kew, https://records.data.kew.org/occurrences/f573f797-57cc-48c7-b444-c6a9b8f085c3 (**A**), Meise Botanic Garden, Meise, Belgium, https://www.botanicalcollections.be/specimen/BR0000031831338 (**B**).

Figs 1 , 2 , 3 , 4 , 5

≡ RhizophoramucronataPoir.var.stylosa (Griff.) A. Schimp., Bot. Mitt. Tropen 3: 92. 1891; Guillaumin in Lecomte et al., Fl. Indo-Chine 2(6): 724. 1920.

##### Type.

Peninsular Malaysia • Malacca (Melaka, Malay), s.d., *W. Griffth s.n.* (lectotype K digital image! [K005752944] (Fig. 5A), designated here; isolectotype: BR digital image! [BR0000031831338] (Fig. 5B).

##### Description.

***Habit*** small evergreen trees, single- to multi-stemmed, 2.5–8 m tall, 10–60 cm GBH. ***Stilt roots*** much-branched, descending from the base of the stem, bearing numerous lenticels. ***Branches and branchlets*** decussate, growing upward at acute angles; branches often with pendulous aerial roots; branchlets terete, glabrous, with conspicuous annular stipular scars and leaf scars at the nodes. ***Bark*** grayish brown or dark brown, varies from smooth, rough or shallowly longitudinally and transversely fissured. ***Stipules*** interpetiolar, in pairs enclosing the young shoot (including the terminal bud and young leaves) and young inflorescences, pale green or green, turning whitish pale green or creamish white before the leaves emerging or before falling off, reddish brown when dry, linear-lanceolate, gradually narrowing towards the apex, 3.5–7.5 cm long, 2.5–9 mm diam. at the base, apex acute, glabrous and caducous, outline in transverse section depressed orbicular basally and suborbicular to orbicular apically, with dense colleters aggregated in a basal band, producing a sticky white exudate; colleters sessile, pale yellow, narrowly conical, 0.5–1.2 mm long, 0.2–0.5 mm diam. at the base, apex obtuse. ***Leaves*** decussate and crowded at the apical part of branchlets, elliptic, 8–16 × 3–8.5 cm, apex mucronate, a short terminal stiff point pale green, turning black before they come off, 2–6 mm long, base cuneate, margin entire, coriaceous, shiny dark green above, pale green below, glabrous on both surfaces, with numerous conspicuous, scattered tiny black cork warts below, midrib pale green (paler than lamina), flattened above, raised below, secondary veins 8–14 on each side, curving towards the margin and connected in distinct loops and united into an intramarginal vein, visible above, obscure below, with intersecondary veins, veinlets reticulate, visible above, obscure below; petioles green, 2–4.8 cm long, 2–4.5 mm diam., glabrous; fresh leaves brittle when crushed; young leaves shiny pale green; mature leaves turning greenish bright yellow and bright yellow before falling off; dry leaves yellowish brown. ***Inflorescences*** axillary, opposite, compound dichasia, dichotomously branched, 4–13-flowered cymes, 5–12.5 cm long; peduncles pale green, 2.3–6 cm long, 2–3.5 mm diam., glabrous. ***Bracts*** 2, pale green, concave, 2.5–4 × 2.7–3.5 mm, apex rounded. ***Bracteoles*** at the base of the flower, pale green, connate at the base, bilobed, 3–5 × 4–6 mm. ***Flowers*** 4-merous; flower buds pale green, turning pale yellow when mature, ovoid or conical-ovoid, 0.9–1.5 cm long, 4–8 mm diam., apex obtuse; fully open flowers 1.2–1.8 cm diam.; sepals 4, erect or patent after anthesis, pale yellow on both sides, triangular, 0.7–1.3 cm × 4–6 mm, apex acute, coriaceous, glabrous; petals 4, creamish white, narrowly elliptic or elliptic, 0.6–1 cm × 1.5–3.5 mm, thin, involute, densely long-villous along margins; stamens 8, 4 antesepalous and 4 antepetalous (each petal enclosing 1 stamen in flower buds); anthers falcate-like, 3.5–8.5 × 1.2–2.8 mm, apex mucronate, triangular in outline in transverse section; filaments very short; free part of the ovary 0.7–1.2 mm long, 1.2–1.8 mm diam.; style 3–5 mm long; stigma 2-lobed; pedicels pale green, 0.6–1 cm long, 2–3 mm diam. ***Fruits*** brownish green, greenish brown or brown, ovoid or conical-ovoid, 2–2.7 cm long, 1–1.7 cm diam. at the base, apex obtuse (before seed germination); obpyriform, 2.2–4 cm long, 1.7–3 cm diam. at the basal part, 0.9–1.7 cm diam. at the apical part (when the hypocotyls nearly come off), roughened surface; persistent sepals reflexed, triangular, 0.9–1.1 cm × 5–8.5 mm; infructescence stalks 2–6 cm long, 2–3 mm diam.; fruit stalks 0.6–1.7 cm long, 3–5 mm diam. ***Seeds*** 1, viviparous. ***Hypocotyls*** green, slightly glossy, cylindrical-clavate, 19.5–40 cm long, 6.5–9 mm diam. at the apical part, 1–1.6 cm diam. at the widest part, 0.8–1.3 cm diam. at the basal part, acute at the basal end, roughened surface, with numerous, scattered lenticels; cotyledonous cylindrical tubes pale green or greenish pale yellow, 0.6–1.2 cm diam. (can be seen when the hypocotyls nearly falling off).

The measurements of the vegetative and reproductive parts of *Rhizophorastylosa* in Thailand are presented in Table 1.

**Table 1.** Measurements of the vegetative and reproductive parts of *Rhizophorastylosa* in Thailand.

Measurements of vegetative and reproductive parts (units)	Sample sizes	Ranges	Mean ± SD
Stipule length (cm)	80	3.5–7.5	5.40 ± 0.92
Stipule diameter at the base (mm)	80	2.5–9.0	4.20 ± 1.58
Colleter length (mm)	50	0.5–1.2	0.72 ± 0.16
Colleter diameter at the base (mm)	50	0.2–0.5	0.35 ± 0.08
Leaf length (cm)	300	8.0–16.0	11.43 ± 1.64
Leaf width (cm)	300	3.0–8.5	5.20 ± 1.02
Leaf length/width ratio	300	1.7–2.9	2.22 ± 0.19
Leaf lamina apex length (mm)	150	2.0–6.0	4.30 ± 1.04
Number of secondary veins each side	300	8–14	11.20 ± 1.68
Petiole length (cm)	300	2.0–4.8	3.25 ± 0.55
Petiole diameter at the middle (mm)	300	2.0–4.5	3.19 ± 0.44
Inflorescence length (cm)	180	5.0–12.5	7.93 ± 1.35
Peduncle length (cm)	180	2.3–6.0	3.80 ± 0.82
Peduncle diameter at the middle (mm)	180	2.0–3.5	2.54 ± 0.34
Number of flowers per inflorescence	180	4–13	6.70 ± 1.57
Mature flower bud length (cm)	200	0.9–1.5	1.25 ± 0.10
Mature flower bud diameter at the basal part (cm)	200	4.0–8.0	6.41 ± 0.82
Mature flower bud length/diameter ratio	200	1.5–2.5	1.97 ± 0.23
Fully open flower diameter (cm)	100	1.2–1.8	1.47 ± 0.15
Sepal length (cm)	200	0.7–1.3	1.01 ± 0.14
Sepal width at the base (mm)	200	4.0–6.0	4.89 ± 0.59
Sepal length/width ratio	200	1.7–2.7	2.06 ± 0.19
Petal length (cm)	200	0.6–1.0	0.79 ± 0.12
Petal width at the middle (cm)	200	1.5–3.5	2.30 ± 0.51
Petal length/width ratio	200	2.2–5.4	3.51 ± 0.62
Anther length (mm)	200	3.5–8.5	6.14 ± 1.14
Anther width at the middle (mm)	200	1.2–2.8	1.70 ± 0.40
Free part of the ovary length (mm)	100	0.7–1.2	0.97 ± 0.12
Free part of the ovary diameter (mm)	100	1.2–1.8	1.47 ± 0.15
Style length (mm)	170	3.0–5.0	3.80 ± 0.52
Pedicel length (cm)	100	0.6–1.0	0.82 ± 0.14
Pedicel diameter (mm)	100	2.0–3.0	2.58 ± 0.26
Fruit length (cm) (hypocotyls nearly come off)	70	2.2–4.0	2.91 ± 0.43
Fruit diameter at the basal part (cm)	70	1.7–3.0	2.04 ± 0.23
Fruit diameter at the apical part (cm)	70	0.9–1.7	1.04 ± 0.17
Persistent sepal length (cm)	150	0.9–1.1	0.98 ± 0.04
Persistent sepal width at the base (mm)	150	5.0–8.5	6.49 ± 0.67
Infructescence stalk length (cm)	60	2.0–6.0	3.26 ± 0.85
Infructescence stalk diameter (mm)	60	2.0–3.0	2.54 ± 0.33
Fruit stalk length (cm)	60	0.6–1.7	1.03 ± 0.25
Fruit stalk diameter (mm)	60	3.0–5.0	3.66 ± 0.39
Hypocotyl length (cm)	75	19.5–40.0	30.01 ± 3.25
Hypocotyl diameter at the apical part (mm)	75	6.5–9.0	7.64 ± 0.65
Hypocotyl diameter at the widest part (cm)	75	1.0–1.6	1.29 ± 0.12
Hypocotyl diameter at the basal part (cm)	75	0.8–1.3	0.99 ± 0.10
Cotyledonous tube diameter (cm)	75	0.6–1.2	0.86 ± 0.14

##### Distribution.

**Figure 6.**
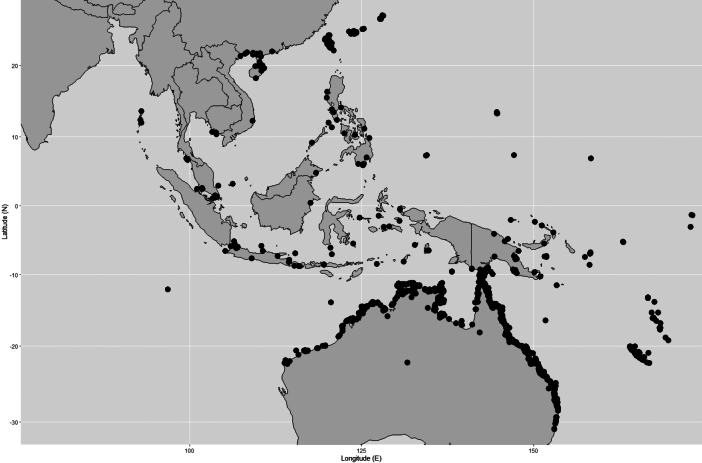
Distribution of *Rhizophorastylosa*. It is widely distributed from India (Andaman and Nicobar Islands), China to Indo-China, and the Pacific Islands. In Thailand, this species is known only from Ko Lidi and Ko Bulon Le within Mu Ko Phetra National Park, located in La-ngu District, and from Ao Talo Wao, Ko Tarutao, in Tarutao National Park, Mueang Satun District, Satun Province. Map: Pichet Chonton & Chatchai Ngernsaengsaruay.

India (Andaman and Nicobar Islands), China (South Guangxi, South Guangdong, Hainan), Taiwan (Taipei), Japan (Ryukyu Islands, also known as Nansei-shoto), Vietnam, Cambodia, Peninsular Thailand, Peninsular Malaysia (also called Malaya), Singapore, Indonesia [Java, Lesser Sunda Islands, Sulawesi (also called Celebes), Moluccas (also called Maluku)], Philippines, Australia (Northern Territory, Queensland, New South Wales, Western Australia), Melanesia (Bismarck Archipelago, New Guinea, Solomon Islands, Vanuatu, Fiji, New Caledonia), Micronesia (Mariana Islands, Caroline Islands, Gilbert Islands), Polynesia (Tuvalu, Tonga, Society Islands) (Fig. 6).

##### Distribution in Thailand.

*Rhizophorastylosa* is known only from Ko Lidi and Ko Bulon Le within Mu Ko Phetra National Park, located in La-ngu District, and from Ao Talo Wao, Ko Tarutao, in Tarutao National Park, Mueang Satun District, Satun Province, Peninsular Thailand (Fig. 6).

##### Habitat and ecology.

*Rhizophorastylosa* typically occurs along coastal areas with muddy-sandy, sandy or sandy-rocky substrates. This species is found at the seaward edge of mangrove forests and is well adapted to grow in open coastal environments, particularly along fairly exposed shores.

The populations of *R.stylosa* observed in Thailand are also located in low intertidal zones characterized by rocky and sandy substrates, similar to the observations reported from the Andaman and Nicobar Islands, India by Ragavan (2015) and Ragavan et al. (2021).

In the Andaman and Nicobar Islands, the species is uncommon and is mostly found in mangroves fringing the open coast, towards the seaward side. It is often found in mid to low intertidal zones and along downstream tidal creeks. The species grows in a variety of habitats, including disturbed mangrove areas. One distinctive niche is its ability to grow along the edges of small coral islands, where it establishes on coral substrates (Ragavan 2015; Ragavan et al. 2021).

##### Phenology.

Flowering, fruiting and viviparous germination more than once, nearly throughout the year.

##### Conservation status.

*Rhizophorastylosa* is widely distributed, ranging from India (Andaman and Nicobar Islands) and China through Indo-China to the Pacific Islands. It is known from numerous localities, with a very large EOO of 52,723,897.41 km^2^ and a relatively large AOO of 1,908 km^2^. In Thailand, however, the species is restricted to Satun Province in the peninsular region, where it has a small EOO of 251.22 km^2^ and a relatively small AOO of 12 km^2^. Despite its limited national distribution, its broad global range, presence in numerous localities, and the absence of significant threats to its survival, we recommend a conservation assessment of Least Concern (LC), in agreement with Ellison et al. (2010).

##### Etymology.

The specific epithet of *Rhizophora stylosa* is a Latin word that refers to its characteristic long style.

##### Vernacular name.

Kongkang phetra (โกงกางเภตรา) (suggested here); Red mangrove, Small stilted mangrove, Spotted mangrove, Stilted mangrove (English).

##### Uses.

To date, there has been no record of its use in Thailand. On Japan’s Iriomote Islands, the traditional dyeing of cotton fabric using tannins extracted from the bark of *Rhizophorastylosa* (Yaeyama hirugi), a technique known as kusaki-zome, remains an important cottage industry. The dye from the outer bark is brownish, while that from the inner bark is reddish (Baba 2004; Baba et al. 2013). On Iriomote and Ishigaki Islands, seedlings of *R.stylosa* are sold in souvenir shops to tourists as ornamentals (Baba et al. 2013). This species is utilized for timber, firewood, and the production of charcoal in the Pacific Island countries (Giesen et al. 2007; Kainuma et al. 2015). In Australia, Aboriginal people use its wood to make boomerangs, spears, and ceremonial items (Giesen et al. 2007).

##### Lectotypification.

*Rhizophorastylosa* was named by Griffith (1854: 665), who cited only the type locality “Hab. Malacca, in littoribus limosis, Pulo Bissar”. However, he did not designate a holotype, nor did he provide a collector number or indicate the herbarium where the material was housed. We have located the specimen *W. Griffth s.n.* from Malacca at K [K005752944] and BR [BR0000031831338], which following Art. 9.6 of the ICN (Turland et al. 2018), must be considered syntypes. William Griffith (1810–1845) was a British colonial physician and botanist who made substantial contributions to the herbarium of the East India Company. His herbarium was originally deposited at LINN, but was later transferred to Kew (K), with significant duplicate specimens housed at BR and CAL (Stafleu and Cowan 1976). Accordingly, we designate the specimen at K [K005752944] as the lectotype, with the specimen at BR [BR0000031831338] being an isolectotype, in accordance with Arts. 9.3 and 9.12 of the ICN (Turland et al. 2018).

##### Notes.

The colors and sizes of the stipules of *Rhizophorastylosa* are pale green or green, turning whitish pale green or creamish white before falling off. They are 3.5–7.5 cm long, 2.5–9 mm diam. at the base. In contrast, the stipules of *R.mucronata* are pale green, reddish pale green, or pale greenish red when young, turning pale yellow or reddish pale yellow before falling off, and are larger, 4.5–12.5 cm in length and 0.5–1.7 cm in diameter at the base. The petioles, peduncles, rachises of inflorescences, and pedicels of *R.stylosa* are usually terete, whereas those of *R.mucronata* are slightly to somewhat flattened.

**Figure 7.**
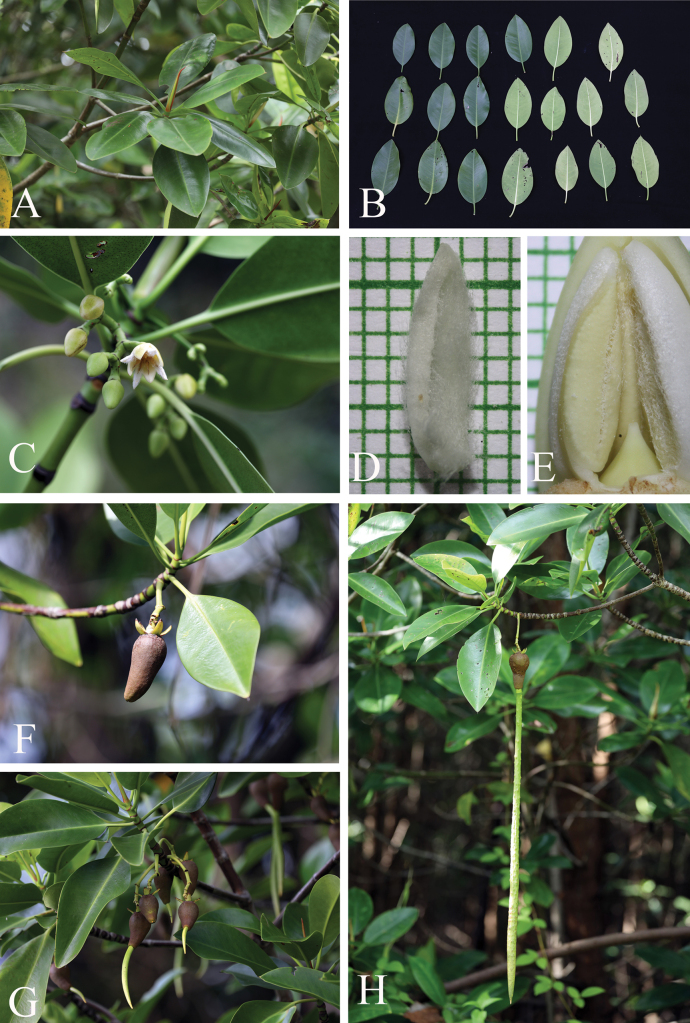
*Rhizophoramucronata*. **A.** Branchlets, leaves and terminal interpetiolar stipule; **B.** Leaves showing adaxial and abaxial surfaces; **C.** Flowering branchlet showing inflorescences with flower buds and fully open flower; **D.** Petal with densely and shorter villous along margins; **E.** Flower bud in longitudinal section showing very short style; **F.** Fruiting branchlet showing leaves and mature fruit with persistent sepals; **G, H.** Fruiting branchlets showing leaves and successive stages in development of hypocotyl. Photos: Chatchai Ngernsaengsaruay (**A–C, F–H**), Pichet Chonton (**D, E**).

Morphologically, *R.stylosa* is closely related to *R.mucronata* (Fig. 7) in its inflorescences and flowers (including flower buds, pedicelled flowers, bracteoles, sepals, and stamens); in the length of the stiff, pointed leaf tips; in the shape and basal diameter of the fruits (when the hypocotyls nearly come off); in the shape and color of the hypocotyls; and in the colors of the cotyledonous cylindrical tubes and colleters. The differences between these two Thai species of *Rhizophora* are presented in Table 2.

**Table 2.** Morphological differences between *Rhizophorastylosa* and *R.mucronata* in Thailand. The morphological characteristics of *R.mucronata* are based on Ngernsaengsaruay et al. (2024).

Morphological characters	*Rhizophorastylosa*	*Rhizophoramucronata*
Habit	Small trees, single- to multi-stemmed, 2.5–8 m tall	Small to large trees, 2–20 (–30) m tall, usually single-stemmed
Stipule colours and sizes	Pale green or green, turning whitish pale green or creamish white before the leaves emerging or before falling off, smaller, 3.5–7.5 cm long and 2.5–9 mm diam at the base	Pale green, reddish pale green or pale greenish red when young, turning pale yellow or reddish pale yellow before the leaves emerging or before falling off, larger, 4.5–12.5 cm long, 0.5–1.7 cm diam at the base
Leaf shapes and sizes	Elliptic, smaller, 8–16 × 3–8.5 cm, widest at the middle part	Ovate or lanceolate-ovate, larger, 13–23.5 × 6–13 cm, widest at the basal part
Petal shapes and hairs	Narrowly elliptic or elliptic, densely and longer villous along margins	Lanceolate-ovate or lanceolate, densely and shorter villous along margins
Free part of the ovary length	Shorter, 0.7–1.2 mm long	Longer, 1.5–3 mm long
Style length	3–5 mm long	Very short
Fruit sizes (ovoid or conical-ovoid, before seed germination)	Smaller, 2–2.7 cm long, 1–1.7 cm diam. at the basal part	Larger, 3.7–5 cm long, 2.1–2.8 cm diam. at the basal part
Fruit sizes (obpyriform, when the hypocotyls nearly come off)	Smaller, 2.2–4 cm long, 1.7–3 cm diam. at the basal part	Larger, 4–7 cm long, 2.4–3.5 cm diam. at the basal part
Hypocotyl length	Shorter, 19.5–40 cm long	Longer, 25–70 cm long

Two distinct populations of *R.stylosa* were found in Mu Ko Phetra National Park, La-ngu District, Satun Province, Peninsular Thailand. Population A is distributed along coastal areas characterized by muddy-sandy substrates, predominantly occupying the seaward edge of the mangrove forest at Ko Lidi, La-ngu Subdistrict. In contrast, Population B occurs along coastal areas with sandy to sandy-rocky substrates, particularly along relatively exposed shorelines at Ko Bulon Le, Pak Nam Subdistrict. Morphological comparisons between the two populations reveal that individuals in Population A (3–8 m) tend to be taller than those in Population B (2.5–5 m). Furthermore, vegetative and reproductive structures, including stipules, leaves, petioles, inflorescences, peduncles, and fruits, are generally larger in Population A. These morphological differences are likely influenced by environmental conditions, suggesting that substrate type and coastal exposure play significant roles in shaping the growth and reproductive traits of *R.stylosa* in this region (Table 3).

**Table 3.** Comparison of the morphological characteristics between two populations of *Rhizophorastylosa* in Mu Ko Phetra National Park, La-ngu District, Satun Province, Thailand. Population A occurs along coastal areas with muddy-sandy substrates, particularly at the seaward edge of mangrove forest at Ko Lidi, La-ngu Subdistrict. Population B occurs along coastal areas with sandy or sandy-rocky substrates, especially along fairly exposed shores at Ko Bulon Le, Pak Nam Subdistrict.

Morphological characters (units)	Sample sizes	*Rhizophorastylosa* Population A		*Rhizophorastylosa* Population B	
		Ranges	Mean ± SD	Ranges	Mean ± SD
Stipule length (cm)	A 30/B 50	4.0–7.5	5.78 ± 1.03	3.5–6.5	5.16 ± 0.77
Stipule diameter at the base (mm)	A 30/B 50	3.0–9.0	5.06 ± 2.04	2.5–6.0	3.68 ± 0.91
Leaf lamina length (cm)	150	9.5–16.0	12.72 ± 1.10	8.0–12.5	10.14 ± 0.94
Leaf lamina width (cm)	150	4.5–8.5	6.01 ± 0.76	3.0–5.5	4.39 ± 0.44
Petiole length (cm)	150	2.5–4.8	3.63 ± 0.44	2.0–4.0	2.88 ± 0.36
Petiole diameter at the middle (mm)	150	2.5–4.5	3.45 ± 0.38	2.0–3.5	2.93 ± 0.31
Inflorescence length (cm)	90	6.0–12.5	8.80 ± 1.18	5.0–9.0	7.05 ± 0.87
Peduncle length (cm)	90	2.8–6.0	4.39 ± 0.65	2.3–4.8	3.22 ± 0.49
Fruit length (cm) (hypocotyls nearly come off)	A 20/B 50	3.0–4.0	3.49 ± 0.31	2.2–3.0	2.68 ± 0.17
Fruit diameter at the basal part (cm)	A 20/B 50	1.9–3.0	2.25 ± 0.31	1.7–2.3	1.95 ± 0.11

##### Additional specimens examined.

**Thailand. Peninsular**: • Satun [Ko Lidi, Mu Ko Phetra National Park, La-ngu Subdistrict, La-ngu District, fl., fr. & vivipary, 22 Apr 2025, *C. Ngernsaengsaruay et al. Rs01-22042025* (BKF); • Ao Phangka, Ko Bulon Le, Mu Ko Phetra National Park, Pak Nam Subdistrict, La-ngu District, fl., fr. & vivipary, 22 Apr 2025, *C. Ngernsaengsaruay et al. Rs02-22042025* (BKF); • ibid., fl., fr. & vivipary, 22 Apr 2025, *C. Ngernsaengsaruay et al. Rs03-22042025* (BKF); Ao Talo Wao, Ko Tarutao, Tarutao National Park, Mueang Satun District, fl., 30 May 2025, *N. Mianmit & N. Kaveethanathum* personal observation with photos; Note. *C. Ngernsaengsaruay et al.* = *C. Ngernsaengsaruay, N. Mianmit, R. Pothitan, V. Jintana, N. Jintana & S. Kamsanor*].

### ﻿A key to the species of *Rhizophora* in Thailand

(Modified from Ngernsaengsaruay et al. 2024)

**Table:** 

1	Inflorescences on leafless branchlets (in the axils of leaf scars), 2-flowered cymes; flowers sessile; flower buds pale green, usually tinged with brown, finely reticulated, broadly ellipsoid or ellipsoid; bracteoles brown, connate, cup-shaped, shallowly lobed; sepals elliptic-oblong, thickly coriaceous; petals flattened, not involute, glabrous; stamens mostly 12; fruits up to 1.8 cm diam. at the basal part (when the hypocotyls nearly come off); hypocotyls dark green or purplish dark green; cotyledonous cylindrical tubes red, dark greenish red or reddish dark green; stipules bearing bright yellow colleters that produce a sticky, clear exudate; the stiff, pointed tips of the leaves usually less than 3 mm long	***Rhizophoraapiculata***
–	Inflorescences axillary, dichotomously branched, many-flowered cymes; flowers pedicelled; flower buds pale green, turning pale yellow when mature, ovoid or conical-ovoid; bracteoles pale green, connate at the base, bilobed; sepals triangular, coriaceous; petals involute, villous along margins; stamens 8; fruits usually more than 1.8 cm diam. at the basal part (when the hypocotyls nearly come off); hypocotyls green; cotyledonous cylindrical tubes pale green or greenish pale yellow; stipules bearing pale yellow colleters that produce a sticky, white exudate; the stiff, pointed tips of the leaves usually more than 3 mm long	**2**
2	Leaves ovate or lanceolate-ovate, larger, 13–23.5 × 6–13 cm; petals lanceolate-ovate or lanceolate, densely and shorter villous along margins; free part of the ovaries, longer, 1.5–3 mm long; style very short; fruits (before seed germination) larger, 3.7–5 cm long, 2.1–2.8 cm diam. at the basal part; fruit (when the hypocotyls nearly come off) larger, 4–7 cm long, 2.4–3.5 cm diam. at the basal part; hypocotyls longer, 25–70 cm long; small to large trees, 2–20 (–30) m tall, usually single-stemmed	***Rhizophoramucronata***
–	Leaves elliptic, smaller, 8–16 × 3–8.5 cm; petals narrowly elliptic or elliptic, densely and longer villous along margins; free part of the ovaries, shorter, 0.7–1.2 mm long; style 3–5 mm long; fruits (before seed germination) smaller, 2–2.7 cm long, 1–1.7 cm diam. at the basal part; fruit (when the hypocotyls nearly come off) smaller, 2.2–4 cm long, 1.7–3 cm diam. at the basal part; hypocotyls shorter, 19.5–40 cm long; small trees, single- to multi-stemmed, 2.5–8 m tall	***Rhizophorastylosa***

### ﻿Anatomical study

#### ﻿Leaf anatomy of *Rhizophorastylosa* in Thailand

The leaf anatomical structure of *Rhizophorastylosa* is composed of five principal tissue layers: adaxial epidermis, hypodermis, palisade mesophyll, spongy mesophyll, and abaxial epidermis. Leaf thickness ranges from 574.11–881.72 μm. A thick cuticular wax layer (cuticle) covers both epidermal surfaces, with the adaxial surface bearing a slightly thicker cuticle than the abaxial surface (Fig. 9).

**Figure 8.**
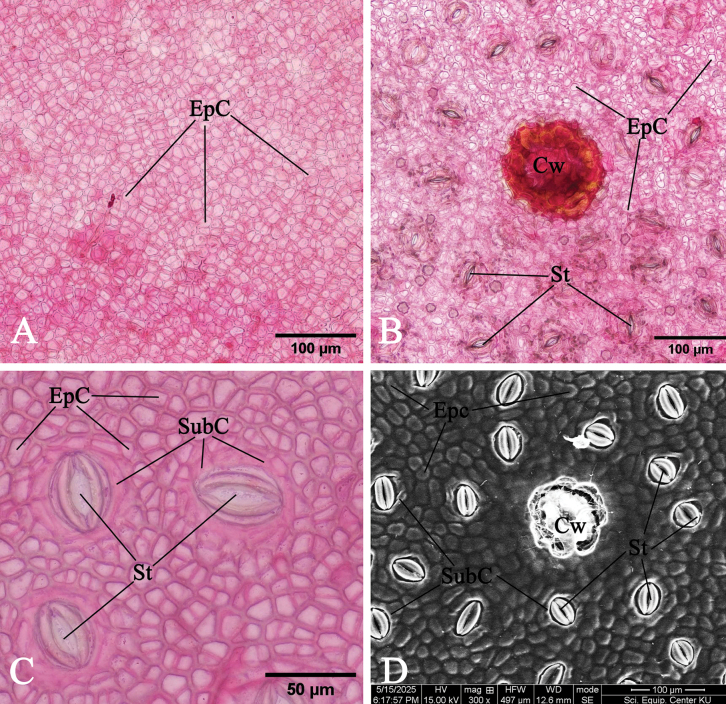
Leaf anatomy of *Rhizophorastylosa*. **A.** Adaxial epidermis (under LM); **B, C.** Abaxial epidermis (under LM); **D.** Abaxial epidermis (under SEM) (Cw = cork wart, EpC = epidermal cells, St = stoma, and SubC = subsidiary cells). Photos: Pichet Chanton & Chatchai Ngernsaengsaruay.

Epidermal cells are polygonal, occasionally trapezoidal with straight anticlinal walls, and are arranged in a single layer on both adaxial and abaxial surfaces. The adaxial epidermis is slightly thicker than the abaxial epidermis (Fig. 8).

**Figure 9.**
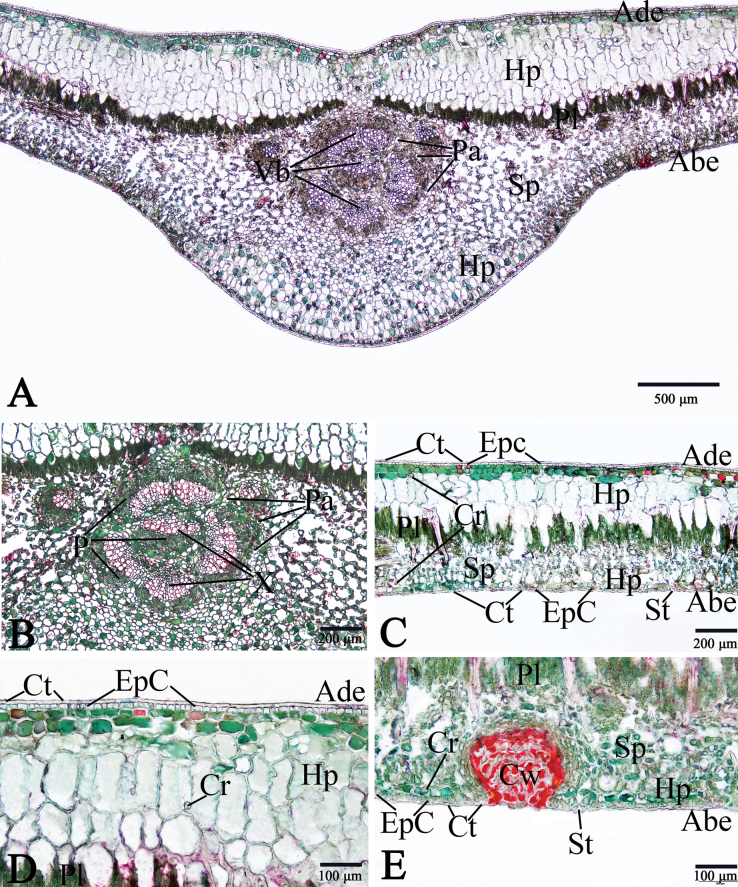
Leaf anatomy of *Rhizophorastylosa*. **A–E.** Transverse section (Abe = abaxial epidermis, Ade = adaxial epidermis, Cr = druse crystals, Ct = cuticle, Cw = cork wart, EpC = epidermal cells, Hp = hypodermis, P = phloem, Pa = parenchyma, Pl = palisade mesophyll, Sp = spongy mesophyll, St = stoma, Vb = vascular bundle, X = xylem). Photos: Pichet Chanton & Chatchai Ngernsaengsaruay.

The stomata of *R.stylosa* are confined to the abaxial leaf surface, indicating that the leaves are hypostomatic.

The stomata are of the sunken cyclocytic type, surrounded by 6–8 subsidiary cells arranged in a concentric ring around each stoma (Fig. 8). Stomatal size ranges from 18.31–30.48 × 9.56–15.21 μm, with a density of 77–153 stomata/mm^2^.

According to previous studies, stomatal density is categorized into three groups: low (<300 stomata/mm^2^), medium (300–500 stomata/mm^2^), and high (>500 stomata/mm^2^). Based on this classification, the stomatal density of *R.stylosa* is considered low. Cork warts are present on the abaxial surface, with a density ranging from 15–52 cork warts percm^2^ and a diameter of 41.57–94.58 μm. These structures consist of densely packed cells, morphologically distinct from other epidermal cells and easily distinguishable from stoma (Fig. 8). *Rhizophorastylosa* has the largest cork wart size among the three species in Thailand. *Rhizophoramucronata* exhibits cork wart sizes ranging from 39.86–82.36 μm, with an average of 61.76 ± 12.83 μm, while *R.apiculata* has the smallest cork wart sizes, ranging from 35.69–75.48 μm, with an average of 54.99 ± 10.64 μm. The data for *R.apiculata* and *R.mucronata* are based on Ngernsaengsaruay et al. (2024).

*Rhizophorastylosa* possesses bifacial leaves. The mesophyll is composed three main tissues: hypodermis, palisade parenchyma, and spongy parenchyma (Fig. 9). The hypodermis consists of relatively large cells arranged in multiple layers beneath the epidermis. These cells are often filled with tannins, which cause them to appear darkly stained. The adaxial surface exhibits a greater average hypodermal thickness compared to the abaxial surface.

Hypodermal cells on the adaxial side are typically subcircular, elliptic, oblong, or rounded polygonal in shape, while those on the abaxial side tend to be broadly elliptic, circular, or rounded polygonal. The average cell size of the hypodermis is also larger on the adaxial surface than on the abaxial surface (Fig. 9).

Druse crystals are present in the hypodermal cells on both the adaxial and abaxial surfaces. The diameter of the druse crystals ranges from 14.84–30.06 μm (Fig. 9).

The palisade parenchyma consists of one to two layers of tightly packed, elongated cells located directly beneath the hypodermis. The spongy parenchyma is composed of five to seven layers of loosely arranged, irregularly shaped cells situated below the palisade parenchyma and above the abaxial epidermis. This tissue exhibits prominent intercellular spaces, forming a characteristic net-like structure (Fig. 9).

Observations of the vascular bundle arrangement in the midrib of *R.stylosa* reveal a complex structure, divided into three distinct regions: abaxial, medullary (central), and adaxial. Each vascular bundle comprises phloem located on the outer side and xylem on the inner side. These vascular bundles are incompletely enclosed by a layer of sclerenchyma cells (Fig. 9).

A comparison of the leaf anatomical characteristics of *R.stylosa* in Thailand with findings from previous studies is presented in Table 4.

**Table 4.** Comparison of the leaf anatomical characteristics of *Rhizophorastylosa* in Thailand with previous studies from other countries: ^[1]^Sheue (2003), ^[2]^Evans et al. (2008), and ^[3]^Putri and Bashri (2023).

Anatomical characters	From the author’s observations	Previous studies
Leaf thickness (μm)	574.11–881.72 (720.53 ± 92.18, n = 20)	571.5 ± 21.3 (Australia)^[1]^ 556.0 ± 31.4 (Singapore)^[1]^
Leaf structure type	Bifacial leaves	Dorsiventral leaves^[1]^ (= bifacial leaves)
Cuticular wax thickness on the adaxial leaf surface (μm)	14.13–34.65 (25.46 ± 5.85, n = 20)	7.04 ± 1.94^[1]^
Cuticular wax thickness on the abaxial leaf surface (μm)	13.27–33.38 (23.80 ± 6.93, n = 20)	5.25 ± 1.23^[1]^
Number of epidermal cell layer on the adaxial leaf surface	1-layered	1-layered^[1]^
Number of epidermal cell layer on the abaxial leaf surface	1-layered	1-layered^[1]^
Epidermal layer thickness on the adaxial leaf surface (μm)	20.40–43.55 (26.97 ± 5.78, n = 20)	–
Epidermal layer thickness on the abaxial leaf surface (μm)	17.25–37.31 (30.05 ± 5.96, n = 20)	–
Epidermal cell shapes on the adaxial leaf surface	Polygonal, sometimes trapezium	Polygonal, 29–35 × 17–25 μm ^[3]^
Epidermal cell shapes on the abaxial leaf surface	Polygonal, sometimes trapezium	Polygonal^[3]^
Stomatal types	Sunken cyclocytic	Cyclocytic^[1]^,
Stomatal density per mm^2^	77–153 (108.91 ± 18.19, n = 100)	61.15 (mean), low density, 9–12 (number of stomata in one field of view, 400× magnification)^[3]^
Stomatal length (μm)	18.31–30.48 (24.65 ± 3.42, n = 100)	49.50 ± 2.70^[1]^,
Stomatal width (μm)	9.56–15.21 (12.20 ± 1.61, n = 100)	43.27 ± 2.27^[1]^,
Stomatal length/width ratio	1.39–3.19 (2.05 ± 0.41, n = 100)	1.14^[1]^
Number of subsidiary cells	6–8 (6.84 ± 0.81, n = 50)	(5–)6–7^[1]^
Cork wart density per cm^2^	15–52 (31.60 ± 9.85, n = 300)	10.1 ± 0.4 (mm^2^)^[2]^
Cork wart density at the apical part per cm^2^	30–52 (41.92 ± 6.11, n = 100)	–
Cork wart density at the middle part per cm^2^	24–40 (31.30 ± 4.88, n = 100)	–
Cork wart density at the basal part per cm^2^	15–28 (21.33 ± 4.07, n = 100)	–
Cork wart diameter (μm)	41.57–94.58 (67.29 ± 15.69, n = 100)	196.00 ± 20.86 (Singapore)^[1]^ 252.00 ± 46.11 (Taiwan)^[1]^
Hypodermal layer thickness on the adaxial leaf surface (μm)	206.41–361.11 (260.49 ± 43.90, n = 20)	–
Hypodermal layer thickness on the abaxial leaf surface (μm)	50.67–110.54 (80.36 ± 18.41, n = 20)	–
Number of hypodermal cell layer on the adaxial leaf surface	4–6-layered (5.25 ± 0.72, n = 20)	2-layered (small-sized) and 3-layered (large-sized)^[1]^, 5- layered^[3]^
Number of hypodermal cell layer on the abaxial leaf surface	2–3-layered (2.45 ± 0.51, n = 20)	1(–3)-layered^[1]^
Hypodermal cell shapes on the adaxial leaf surface (μm)	Subcircular, elliptic, oblong or rounded polygonal	Rounded hexagon ^[3]^
Hypodermal cell length on the adaxial leaf surface (μm)	31.90–99.67 (73.12 ± 20.65, n = 20)	49–73^[3]^
Hypodermal cell width on the adaxial leaf surface (μm)	29.69–73.84 (53.55 ± 12.55, n = 20)	34–46^[3]^
Hypodermal cell shapes on the abaxial leaf surface (μm)	Broadly elliptic, circular or rounded polygonal	–
Hypodermal cell length on the abaxial leaf surface (μm)	18.78–47.27 (33.53 ± 6.92, n = 20)	–
Hypodermal cell width on the abaxial leaf surface (μm)	19.61–46.08 (31.32 ± 6.21, n = 20)	–
Crystal location, type and diameter (μm)	Druse (in hypodermal cells), 14.84–30.06 (22.62 ± 3.98, n = 20)	–
Number of palisade cell layer	1–2-layered (1.40 ± 0.50, n = 20)	1–4-layered (upper), 0–1-layered (lower)^[1]^
Number of spongy cell layer	5–7-layered (6.05 ± 0.76, n = 20)	9–10-layered (Australia), 7–8-layered (Singapore)^[1]^

### ﻿Palynological study

#### ﻿Pollen morphology of *Rhizophorastylosa* in Thailand

The pollen grains of *Rhizophorastylosa* are monads, isopolar, radially symmetrical, and tricolporate. They exhibit either a prolate spheroidal or oblate spheroidal shape, with a P/E ratio ranging from 0.88–1.15. In polar view (amb), the outline is subcircular or rounded triangular.

**Figure 10.**
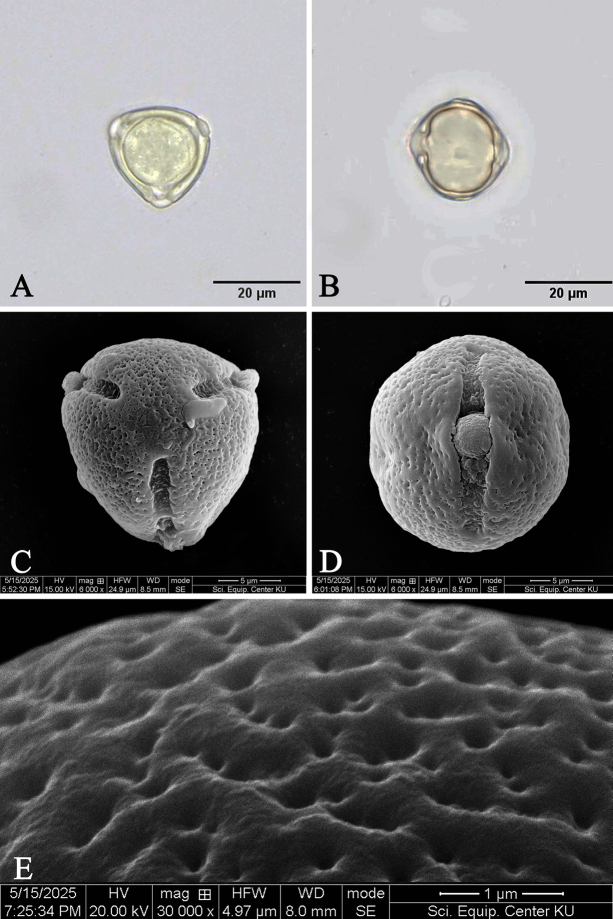
LM and SEM micrographs of pollen grains of *Rhizophorastylosa*. **A.** Tricolporate aperture and rounded-triangular shape in polar view; **B.** Colporate aperture in equatorial view; **C.** Tricolporate aperture and rounded-triangular shape in polar view; **D.** Colporate aperture in equatorial view; **E.** Exine sculpturing, under LM (**A, B**), under SEM (**C–E**). Photos: Pichet Chanton & Chatchai Ngernsaengsaruay.

The pollen grains are small in size, with the polar axis measuring 17.93–22.36 μm and the equatorial axis 16.79–22.94 μm. The colpus length ranges from 12.92–20.40 μm, and the colpus width from 2.35–3.85 μm. The exine thickness ranges from 0.83–2.43 μm., and the exine sculpturing is reticulate (Fig. 10). *Rhizophorastylosa* has the largest pollen size among the three species in Thailand. *Rhizophoramucronata* exhibits pollen sizes along the polar axis ranging from 12.37–18.49 μm, with an average of 15.77 ± 1.58 μm, while *R.apiculata* has the smallest pollen size along the polar axis, ranging from 11.16–13.50 μm, with an average of 12.36 ± 0.78 μm. The data for *R.apiculata* and *R.mucronata* are based on Ngernsaengsaruay et al. (2024).

A comparison of the pollen morphology of *Rhizophorastylosa* in Thailand with previous studies is presented in Table 5.

**Table 5.** Comparison of the pollen morphology of *Rhizophorastylosa* in Thailand with previous studies from other countries: ^[1]^Mao et al. (2012) and ^[2]^Mohd-Arrabé and Noraini (2013).

Pollen characters	From the author’s observations	Previous studies
Polar axis [P] length (μm)	17.93–22.36 (20.59 ± 1.35, n = 30)	19.9 (20.5) 22.7 × 21.3 (22.4) 24.7^[1]^; 17.29 (19.17) 21.27^[2]^
Equatorial axis [E] length (μm)	16.79–22.94 (20.08 ± 1.64, n = 30)	16.61 (18.62) 20.46^[2]^
P/E ratio	0.88–1.15 (1.03 ± 0.07, n = 30)	1.03^[2]^
Pollen size classes	Small	–
Pollen shapes	Prolate spheroidal or oblate spheroidal	Subprolate, circular to subcircular^[1]^; prolate-speroidal^[2]^
Pollen aperture	Tricolporate	Tricolporate^[1], [2]^
Colpus length (μm)	12.92–20.40 (16.48 ± 2.15, n = 30)	13.4^[1]^
Colpus width (μm)	2.35–3.85 (3.21 ± 0.37, n = 30)	–
Exine thickness (μm)	0.83–2.43 (1.54 ± 0.39, n = 30)	1.5^[1]^; 0.79 (0.95) 1.24^[2]^
Exine sculpturing	Reticulate	Scabrate to slightly granulate to irregularly pitted^[1]^; perforate^[2]^

## Supplementary Material

XML Treatment for Rhizophora stylosa

## ﻿Additional information

### Author ORCIDs

Chatchai Ngernsaengsaruay https://orcid.org/0000-0002-7131-976X

Nittaya Mianmit https://orcid.org/0000-0002-1295-8078

Vipak Jintana https://orcid.org/0000-0002-7703-7288

Pichet Chanton https://orcid.org/0009-0001-7325-6109

### Data availability

All of the data that support the findings of this study are available in the main text.
